# Supplementary material for: Lower Nerve Growth Factor Levels in Major Depression and Suicidal Behaviors: Effects of Adverse Childhood Experiences and Recurrence of Illness
Source: Brain Sci. 2023 Jul 18;13(7):1090. doi: 10.3390/brainsci13071090 (PMC10377511; doi:10.3390/brainsci13071090)
Supplement: Supplementary file 1 [file brainsci-13-01090-s001.zip › brainsci-2459729-supplementary.pdf]

ELECTRONIC SUPPLEMENTARY FILE

Lowered nerve growth factor in major depression and suicidal behaviors: effects of adverse childhood experiences and recurrence of illness

(1,2,3,4) Michael Maes, (1) Muanpetch Rachayon; (1,5) Ketsupar Jirakran; (6,7) Pimpayao Sodsai; (1) Atapol Sughondhabirom

ESF, Table S1. Overview of the cytokines, chemokines, and growth factors measured in the current study

Protein abbreviations	Gene Symbol	Protein name	Number of samples with concentrations lower than the sensitivity (%)
IL-1β	IL1B	Interleukin-1β	0
IL-2	IL2	Interleukin-2	0
IL-6	IL6	Interleukin-6	0
CXCL8 / IL-8	CXCL8	C-X-C motif chemokine ligand 8 (IL-8)	0
IL-12	IL12	Interleukin-12	0.5
IL-17	IL17	Interleukin-17	0
CCL11	CCL11	Eotaxin	0
G-CSF	CSF3	Granulocyte Colony Stimulating Factor, Colony Stimulating Factor 3 (Granulocyte)	0
GM-CSF	CSF2	Granulocyte-macrophage colony-stimulating factor, Colony-stimulating factor 2	0
IFN-γ	IFNG	Interferon-γ	0
CXCL10 / IL-10	CXCL10	C-X-C motif chemokine ligand 8, Interferon gamma-induced protein 10 (IP10)	0
CCL2 / MCP1	CCL2	C-C Motif Chemokine Ligand 2 (MCP1)	0
CCL3 / MIP-1α	CCL3	Macrophage inflammatory protein-1 alpha, C-C Motif Chemokine Ligand 3	0
CCL4 / MIP-1β	CCL4	C-C Motif Chemokine Ligand 4, Macrophage Inflammatory Protein 1-Beta, Lymphocyte Activation Gene 1 Protein	0
CCL5 /RANTES	CCL5	C-C Motif Chemokine Ligand 5, Regulated Upon Activation, Normally T-Expressed, And Presumably Secreted	0
TNF-α	TNF	Tumor Necrosis Factor-Alpha	0
Basic FGF	FGF2	Fibroblast growth factor 2, Basic fibroblast growth factor	0

PDGF	PDGFA	Platelet Derived Growth Factor Subunit A	0
VEGF	VEGFA	Vascular Endothelial Growth Factor	0
BNGF	NGF	β-nerve growth factor (NGF)	0
SCF	KITLG	Stem cell factor (SCF) or Kit ligand (KITLG)	0
SCGF	CLEC11A	Stem cell growth factor (SCGF) or C-type lectin domain family 11 member A (CLEC11A)	0
M-CSF	CSF1	Macrophage colony-stimulating factor (M-CSF) or colony stimulating factor 1 (CSF1)	0
HFG or SF	HGF	Hepatocyte growth factor (HGF) or scatter factor (SF)	0

Adapted from:

Maes M, Rachayon M, Jirakran K, Sodsai P, Klinchanhom S, Gałecki P, Sughondhabirom A, Basta-Kaim A. The Immune Profile of Major Dysmood Disorder: Proof of Concept and Mechanism Using the Precision Nomothetic Psychiatry Approach. Cells. 2022 Mar 31;11(7):1183. doi: 10.3390/cells11071183. PMID: 35406747; PMCID: PMC8997660.

Kalayasiri R, Dadwat K, Supaksorn T, ,Sirivichayakul S, Maes M. Methamphetamine (MA) use, MA dependence, and MA-induced psychosis are associated with increasing aberrations in the compensatory immunoregulatory system and interleukin-1α and CCL5 levels. medRxiv 2023.03.26.23287766; doi: https://doi.org/10.1101/2023.03.26.23287766

ESF Table S2. Description of the immune and growth factor profiles used in this study

Immune Profile	Members
**Neurotoxicity (NT)**	IL-1β, IL-6, TNF-α, CXCL8, CCL3, IL-2, IFN-γ, IL-12, IL-17, CXCL10, CCL11, CCL5, CCL2
**Growth factors (GF)**	FGF, PDGF, VEGF
**zNGF-zNT**	z NGF – z Neurotoxicity
**zNGF-zGF**	z NGF – z growth factors
**zNGF–z(NT+GF)**	z NGF – z (NT+GF)

NGF: nerve growth factor

Maes M, Rachayon M, Jirakran K, Sodsai P, Klinchanhom S, Gałecki P, Sughondhabirom A, Basta-Kaim A. The Immune Profile of Major Dysmood Disorder: Proof of Concept and Mechanism Using the Precision Nomothetic Psychiatry Approach. Cells. 2022 Mar 31;11(7):1183. doi: 10.3390/cells11071183. PMID: 35406747; PMCID: PMC8997660.

Kalayasiri R, Dadwat K, Supaksorn T, ,Sirivichayakul S, Maes M. Methamphetamine (MA) use, MA dependence, and MA-induced psychosis are associated with increasing aberrations in the compensatory immunoregulatory system and interleukin-1α and CCL5 levels. medRxiv 2023.03.26.23287766; doi: https://doi.org/10.1101/2023.03.26.23287766

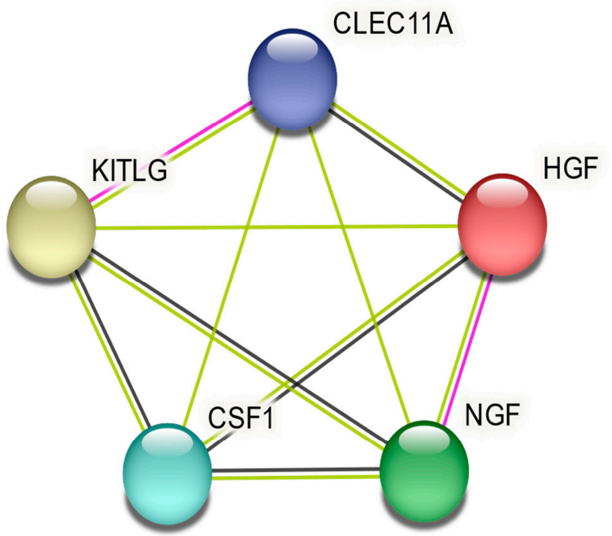

ESF Figure S1. Network of 5 selected growth factors. The PPI network of the 5 growth factors consists of 5 nodes and 10 edges, which is greater than the expected number of edges (n = 0) with a p-enrichment value of 4.19 e-11. The growth factor network has an average node degree of 4 and an average local clustering coefficient of 1. See ESF Table S1 for explanation of the gene IDs. See ESF Table S1 for gene IDs.

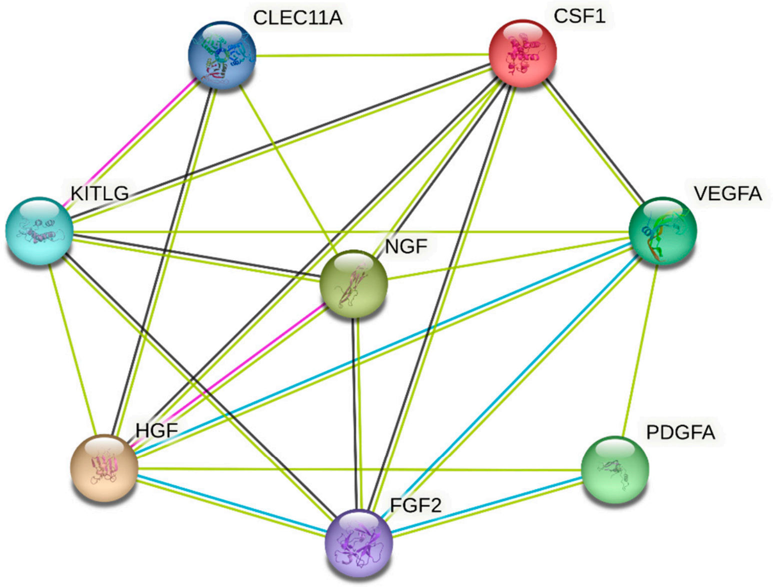

ESF Figure S2. Network of 8 selected growth factors. The PPI network of the 8 growth factors consists of 8 nodes and 22 edges, which is greater than the expected number of edges (n = 2) with a p-enrichment value of 3.22 e-15. The growth factor network has an average node degree of 5.5 and an average local clustering coefficient of 0.864. See ESF Table S1 for explanation of the gene IDs.

ESF Table S3. Measurements of the growth factors in healthy controls (HC), simple depression (SDMD) and major dysmood disorder (MDMD)

Estimates

				95% Confidence Interval	
Dependent Variable	MajorDysmoodDisorder	Mean	Std. Error	Lower Bound	Upper Bound
NGF	HC=1	.083[a]	.212	-.344	.509
	SDMD=2	-.613[a]	.283	-1.184	-.043
	MDMD=33	-.900[a]	.211	-1.325	-.475
SCF	1	.008[a]	.237	-.469	.484
	2	-.043[a]	.317	-.681	.594
	3	-.392[a]	.236	-.867	.084
SCGF	1	.032[a]	.231	-.434	.497
	2	-.071[a]	.309	-.693	.552
	3	-.294[a]	.230	-.757	.170
HGF	1	-.011[a]	.236	-.486	.464
	2	-.214[a]	.316	-.850	.422
	3	-.212[a]	.235	-.686	.262
MCSF	1	.104[a]	.228	-.354	.563
	2	.357[a]	.305	-.256	.971
	3	-.077[a]	.227	-.534	.381
NT	1	.014[a]	.216	-.421	.448
	2	.152[a]	.288	-.429	.733
	3	.941[a]	.215	.508	1.374

NGF/NT	1	.049[a]	.193	-.340	.438
	2	-.549[a]	.258	-1.069	-.029
	3	-1.320[a]	.192	-1.707	-.932
GF	1	-.025[a]	.216	-.460	.410
	2	.388[a]	.289	-.194	.969
	3	.987[a]	.215	.554	1.421
NGF/GF	1	.069[a]	.197	-.329	.466
	2	-.639[a]	.264	-1.171	-.108
	3	-1.205[a]	.197	-1.602	-.809
NGF/NT+GF	1	.060[a]	.194	-.330	.449
	2	-.605[a]	.259	-1.126	-.083
	3	-1.291[a]	.193	-1.680	-.902

a. Covariates appearing in the model are evaluated at the following values: Gender = 1.66, Age = 30.66.

Pairwise Comparisons

Dependent Variable	(I) MajorDysmoodDisorder	(J) MajorDysmoodDisorder	Mean Difference (I-J)	Std. Error	Sig.[b]	95% Confidence Interval for Difference[b]	
						Lower Bound	Upper Bound
NGF	1	2	.696	.363	.062	-.036	1.428
		3	.982*	.303	.002	.372	1.592
	2	1	-.696	.363	.062	-1.428	.036
		3	.287	.350	.417	-.418	.991
	3	1	-.982*	.303	.002	-1.592	-.372
		2	-.287	.350	.417	-.991	.418
SCF	1	2	.051	.406	.901	-.766	.868

		3	.399	.338	.244	-.282	1.081
	2	1	-.051	.406	.901	-.868	.766
		3	.348	.391	.378	-.439	1.135
	3	1	-.399	.338	.244	-1.081	.282
		2	-.348	.391	.378	-1.135	.439
SCGF	1	2	.102	.396	.798	-.696	.900
		3	.325	.330	.330	-.340	.990
	2	1	-.102	.396	.798	-.900	.696
		3	.223	.381	.562	-.545	.991
	3	1	-.325	.330	.330	-.990	.340
		2	-.223	.381	.562	-.991	.545
HGF	1	2	.203	.405	.618	-.612	1.018
		3	.201	.337	.554	-.478	.881
	2	1	-.203	.405	.618	-1.018	.612
		3	-.002	.390	.996	-.787	.783
	3	1	-.201	.337	.554	-.881	.478
		2	.002	.390	.996	-.783	.787
MCSF	1	2	-.253	.391	.520	-1.040	.534
		3	.181	.326	.581	-.475	.837
	2	1	.253	.391	.520	-.534	1.040
		3	.434	.376	.255	-.323	1.191
	3	1	-.181	.326	.581	-.837	.475
		2	-.434	.376	.255	-1.191	.323
NT	1	2	-.138	.370	.710	-.883	.607
		3	-.927*	.308	.004	-1.548	-.306
	2	1	.138	.370	.710	-.607	.883

		3	-.789*	.356	.032	-1.506	-.071
	3	1	.927*	.308	.004	.306	1.548
		2	.789*	.356	.032	.071	1.506
NGF/NT	1	2	.598	.331	.078	-.069	1.265
		3	1.369*	.276	<.001	.813	1.925
	2	1	-.598	.331	.078	-1.265	.069
		3	.771*	.319	.020	.129	1.413
	3	1	-1.369*	.276	<.001	-1.925	-.813
		2	-.771*	.319	.020	-1.413	-.129
GF	1	2	-.413	.370	.271	-1.158	.333
		3	-1.012*	.309	.002	-1.634	-.391
	2	1	.413	.370	.271	-.333	1.158
		3	-.599	.356	.100	-1.317	.118
	3	1	1.012*	.309	.002	.391	1.634
		2	.599	.356	.100	-.118	1.317
NGF/GF	1	2	.708*	.339	.042	.026	1.390
		3	1.274*	.282	<.001	.706	1.842
	2	1	-.708*	.339	.042	-1.390	-.026
		3	.566	.326	.089	-.091	1.222
	3	1	-1.274*	.282	<.001	-1.842	-.706
		2	-.566	.326	.089	-1.222	.091
NGF/NT+GF	1	2	.664	.332	.052	-.005	1.333
		3	1.350*	.277	<.001	.793	1.908
	2	1	-.664	.332	.052	-1.333	.005
		3	.686*	.320	.037	.043	1.330
	3	1	-1.350*	.277	<.001	-1.908	-.793

		2	-.686*	.320	.037	-1.330	-.043

Based on estimated marginal means
*. The mean difference is significant at the .05 level.

NGF: nerve growth factor; SCF: stem cell factor; SCGF: stem cell growth factor; HGF: hepatocyte growth factor; M-CSF: macrophage colony stimulating factor.

GF: composite built using platelet-derived growth factor, vascular endothelial growth factor, and fibroblast growth factor; neurotoxicity; see ESF Table S2 for computation.
